# Investigation of the effect of a virtual reality-based imagery training model on muscle activation in athletes

**DOI:** 10.3389/fpsyg.2025.1553327

**Published:** 2025-02-27

**Authors:** Fatih Bedir, Deniz Bedir, Hasan Hüseyin Yılmaz, Fatih Ağduman, İlhan Şen, Fatih Kıyıcı, Onur Erdem Korkmaz, Mustafa Onur Yıldız, Erkan Çelik

**Affiliations:** ^1^Faculty of Sport Science, Atatürk University, Erzurum, Türkiye; ^2^Faculty of Sport Science, Erzurum Technical University, Erzurum, Türkiye; ^3^Sports Sciences Application and Research Center, Atatürk University, Erzurum, Türkiye; ^4^Faculty of Engineering, Atatürk University, Erzurum, Türkiye; ^5^Faculty of Medicine, Samsun University, Erzurum, Türkiye

**Keywords:** virtual reality, imagery training, PETTLEP, muscle activation, peak performance

## Abstract

**Introduction:**

In the field of sports psychology, imagery training plays a significant role in enhancing athletes’ mental preparation and optimizing sports performance. This study aims to investigate the effects of the Virtual Reality-Based Imagery (VRBI) training model on muscle activation and kinesthetic motor imagery skills in athletes. Specifically, the study compares the VRBI model with traditional imagery methods, including Visual Motor Behavior Practice (VMBP) and Video Modeling (VM), to determine its effectiveness in improving neuromuscular responses.

**Methods:**

A quasi-experimental design with repeated measures was employed, involving 30 bodybuilders and fitness athletes who were randomly assigned to VRBI, VMBP+VM, and control groups. Muscle activation was measured using surface electromyography (sEMG) across a 12-week intervention period. The intervention protocols included progressive relaxation, video modeling, and imagery exercises tailored to enhance kinesthetic motor imagery skills.

**Results:**

The results revealed that the VRBI model significantly increased muscle activation levels and kinesthetic motor imagery skills compared to both the VMBP+VM and control groups (*p* < 0.01). Notably, athletes in the VRBI group achieved peak muscle activation one week earlier than those in the VMBP+VM group, demonstrating a faster adaptation process. Additionally, VRBI training led to a more substantial improvement in imagery skills, suggesting its superiority in mental training interventions.

**Discussion and conclusion:**

The VRBI model offers a more effective approach to enhancing muscle activation and kinesthetic motor imagery skills in athletes. These findings highlight the potential of VRBI as a valuable tool for optimizing sports performance and accelerating peak performance achievement.

## Introduction

In today’s competitive sports environment, delivering high-level performance is a primary expectation from athletes (Tokdemir, 2011). Sports performance is influenced by various factors, including physical fitness, technical skills, and psychological readiness (Bayraktar and Kurtoğlu, 2009). While modern training systems have minimized physical and physiological differences among athletes, psychological factors have become crucial for gaining a competitive edge (Chaouachi et al., 2009). Psychological skills, such as goal setting, imagery, arousal regulation, concentration, and mental preparation, play a vital role in enhancing sports performance (Beauchamp et al., 1996; Greenspan and Feltz, 1989; Mullen and Copper, 1994; Smith and Christensen, 1995; Thelwell and Greenlees, 2001). These skills can be developed through training, positively impacting both training outcomes and competitive performance (Weinberg and Gould, 2007; Vealey, 1988).

Among these, imagery is one of the most frequently used psychological techniques due to its practical application and direct influence on performance. It involves mental visualization of experiences, helping athletes improve focus, build confidence, and manage competition-related stress (Gould et al., 2014; Weinberg and Gould, 2019).

### Imagery

Imagery is the mental creation of experiences using various senses, including auditory, visual, tactile, olfactory, gustatory, kinesthetic, and organic sensations (Dickstein and Deutsch, 2007). These experiences, rooted in memory, can be recalled and modified as needed (Beşiktaş, 2012). Widely applied in language development, motivation, motor skill learning, and rehabilitation (Suica et al., 2018; Gammage et al., 2000), imagery enhances sports performance, skill acquisition, and emotional regulation (Fortes et al., 2019; Williams, 2019; Bedir and Erhan, 2021), while also aiding rehabilitation by reducing recovery time and muscle atrophy after injuries (Dickstein and Deutsch, 2007; Gregg et al., 2010).

### Motor imagery

Motor imagery (MI) is the mental representation of movement without actual bodily motion (Guillot and Collet, 2005; Solodkin et al., 2004). It involves mentally rehearsing specific motor actions in working memory without producing physical movement (Guillot and Collet, 2010). MI is categorized into kinesthetic motor imagery, which focuses on the ‘feeling’ of joint movements and muscle activations, and visual motor imagery, which involves mentally visualizing movements (Stecklow et al., 2010; Paris-Alemany et al., 2019). For instance, a tennis player may visualize serving the ball (visual MI) or mentally experience the sensations of body balance and muscle tension during the serve (kinesthetic MI).

### Theories of imagery

Research on imagery has attempted to explain the imagery-performance relationship through various theories. Although many theories have been developed regarding this relationship, the most popular today in terms of muscle activation is the psycho-neuro-muscular theory.

#### Psycho-neuro-muscular theory

Also known as the Information Processing Theory, this theory suggests that imagery involves recalling and recreating past experiences, generating neuromuscular responses similar to actual movements (Carpenter, 1875; Morris et al., 2005; Konter, 1999). Based on Carpenter’s ‘ideo-motor’ principle, it proposes two key assumptions: first, imagining physical actions triggers weak, localized muscle activity, known as the ‘Carpenter effect’ (Altıntaş and Akalan, 2008); second, this activity provides kinesthetic feedback, aiding skill preparation (Lavallee et al., 2012; Moran, 2013). Jacobson (1934) observed low-intensity muscle contractions during imagined movements, mirroring real actions but at reduced intensity. Despite lower activation levels, this process effectively enhances motor planning in the cortex (Konter, 1999).

### Effective imagery

The human brain struggles to distinguish between vividly imagined situations and reality, as both activate similar neural pathways and neurochemical mechanisms (Suinn, 1986; Cox and Cox, 2002; Martens, 1987; Weinberg and Gould, 2007). Imagery generates stimuli in the central nervous system, preparing muscles for actual performance through electrical responses, enhancing athletic readiness (Murru and Martin Ginis, 2010; Tod et al., 2011; Beşiktaş, 2012; Acevedo and Ekkekakis, 2006). While imagery generally improves performance, its effectiveness varies based on an athlete’s ability to create clear, realistic mental images (Cox and Cox, 2002; Bedir and Erhan, 2021). To enhance imagery quality, engaging all senses is crucial (Bedir and Erhan, 2021). Vivid, multi-sensory imagery positively impacts sports performance (Acevedo and Ekkekakis, 2006; Wilson et al., 2009; Mouratidis et al., 2008). The PETTLEP model (Holmes and Collins, 2001) supports this by focusing on physical, environmental, task-related, temporal, learning, emotional, and perspective aspects to strengthen the functional equivalence between imagined and real actions (Harris and Hebert, 2015). PETTLEP-based interventions have shown positive effects in sports like athletics, gymnastics, and strength training (Lebon et al., 2010; Wright and Smith, 2007).

### Studies on imagery related to muscle activation and muscle strength

Sport psychologists use techniques like focused attention, preparatory arousal, imagery, self-efficacy, self-talk, and relaxation to enhance motor performance without physical movement (Tod et al., 2003, 2015). These methods effectively support strength development in both athletes and non-athletes (Shelton and Mahoney, 1978; Whelan et al., 1990; Gould et al., 1980).

Motor imagery, widely used in sports (Cumming and Williams, 2012) and rehabilitation (Braun et al., 2013; Caligiore et al., 2017), aids recovery from conditions like Parkinson’s disease, immobilization, stroke, and orthopedic surgeries (Tamir et al., 2007; Newsom et al., 2003; Lee et al., 2015; Marusic et al., 2018). Research links motor imagery to increased strength performance, with greater benefits observed when combined with physical training (Tod et al., 2015; Manochio et al., 2015). Additionally, kinesthetic motor imagery has shown superior effects on muscle strength compared to visual motor imagery (Yao et al., 2013; Slimani et al., 2016).

### Imagery trainings

**Figure 1:**
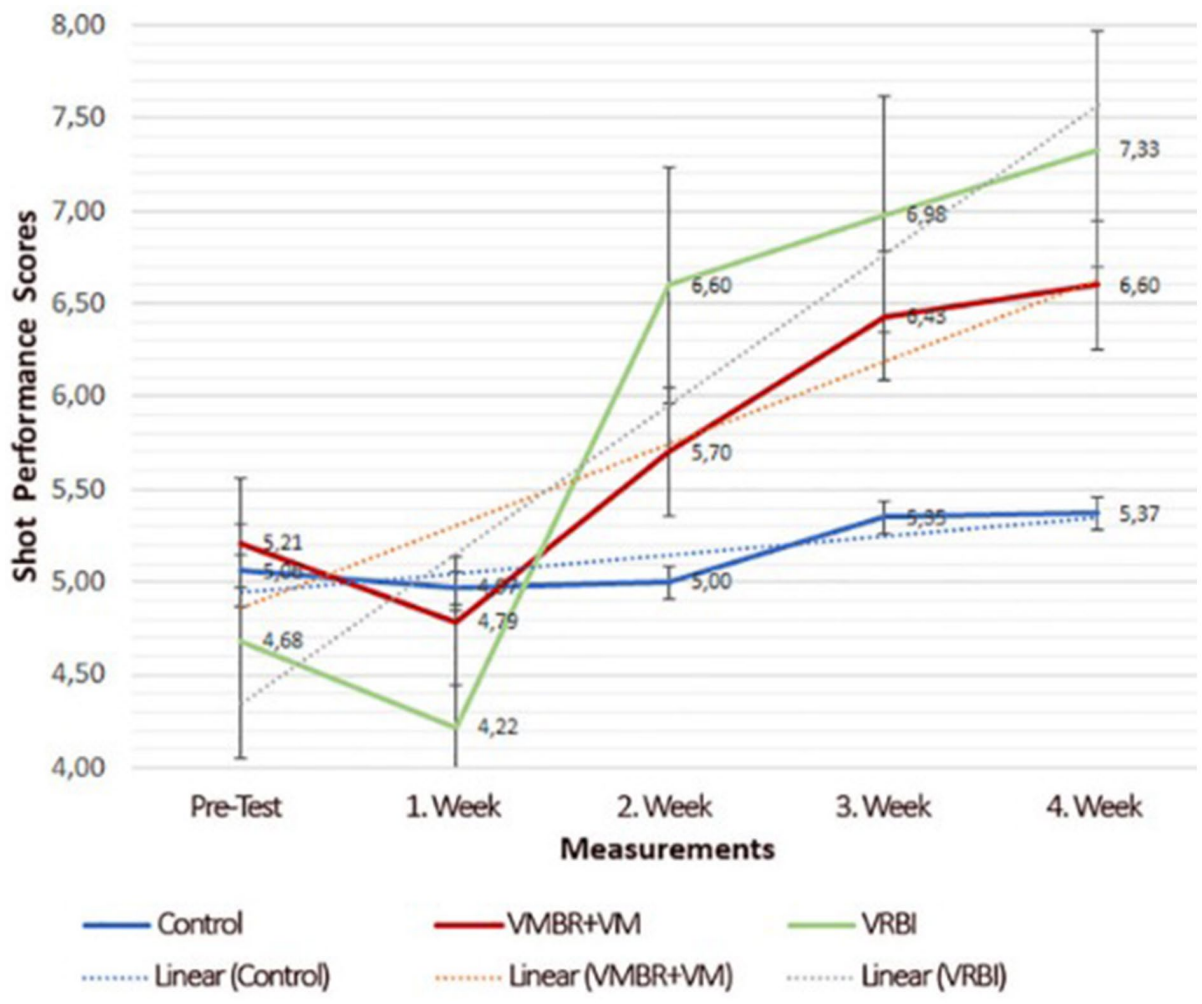
The weekly results graph for shot-delivery performance scores by group (Bedir and Erhan, 2021).

**Figure 2:**
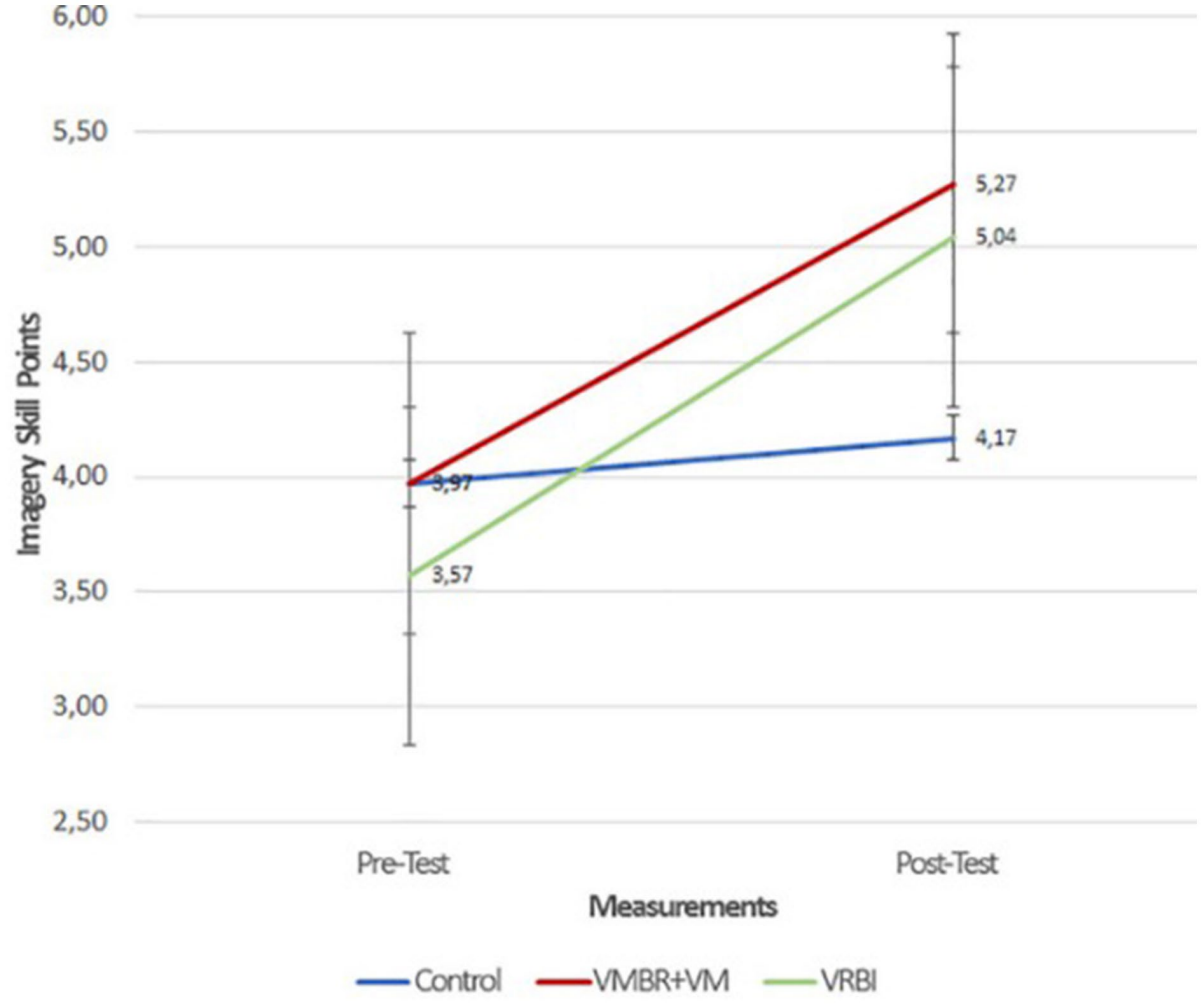
The pre- and post-test results graph for imagery skill scores by group (Bedir and Erhan, 2021).

With the growing popularity of imagery in sports, research has focused on enhancing its effectiveness. Key factors influencing imagery quality include an athlete’s skills, experience level, and training methods (Hall, 2001; Arvinen-Barrow et al., 2007; Gregg and Hall, 2006; Robin et al., 2007; Macintyre et al., 2013). Techniques like Visuo Motor Behavior Rehearsal (VMBR) and Video Modeling (VM) are designed to produce realistic imagery. VMBR combines relaxation with visualization in stressful scenarios to simulate real performance experiences (Suinn, 1972a,b; Noel, 1980; Suinn, 1986), while VM relies on observational learning to encode motor skills through demonstrations (Bandura, 1986; Cumming et al., 2005; Buck et al., 2016). Both are effective independently or combined (Bedir and Erhan, 2021). A more recent advancement is the Virtual Reality-Based Imagery (VRBI) model, which creates immersive environments using 3D technology to enhance sensory engagement (Üzümcü et al., 2018; Kim et al., 2009). VRBI integrates PETTLEP components, promoting mental rehearsal through progressive relaxation, 3D performance observation, imagery exercises, and real-life execution (Bedir and Erhan, 2021). In this context, Bedir and Erhan (2021) compared the VMBR+VM and VRBI models concerning sports performance and motor imagery skills, revealing the advantages of the VRBI model (Figures 1, 2).

In elite athletes, where physical and physiological characteristics are nearly identical, psychological factors play a critical role in gaining a competitive advantage (Chaouachi et al., 2009). High levels of psychological skills, such as goal setting, imagery, arousal regulation, and concentration, can significantly influence performance outcomes (Beauchamp et al., 1996; Greenspan and Feltz, 1989; Mullen and Copper, 1994; Smith and Christensen, 1995; Thelwell and Greenlees, 2001). To enhance these skills, new psychological training models have been developed, with the Virtual Reality-Based Imagery (VRBI) model emerging as a promising and potentially effective method (Suinn, 1972a,b; Bandura, 1986; Bedir and Erhan, 2021). The effectiveness of any training model depends on variables such as intensity, duration, and frequency. Overlooking these factors can limit performance gains, making it essential to identify the optimal conditions for athletic improvement. This principle also applies to imagery training, where determining the duration required to achieve peak neural and muscular activation is crucial. Additionally, common sports injuries often necessitate immobilization, leading to muscle weakness even after short periods (Bayraktar, 2011; Zijdewind et al., 2003). Kinesthetic motor imagery has shown promise in mitigating these effects and supporting muscle strength during recovery (Gözaçan Karabulut et al., 2022). Understanding the effects of VRBI on muscle activation could provide valuable insights not only for athletic performance but also for medical fields such as stroke rehabilitation, cerebral palsy, and orthopedic recovery.

This study aims to evaluate the effects of VRBI training on muscle activation and kinesthetic motor imagery skills. By comparing VRBI with traditional imagery methods, we seek to determine its effectiveness in enhancing athletic performance and supporting rehabilitation. The study also addresses the gap in current literature regarding VRBI’s role in optimizing training protocols and identifying the duration required for peak neuromuscular activation. To investigate these effects, the leg extension exercise, commonly used for strength development, was incorporated into a virtual reality setting. VRBI training not only simulates real-life movements but also reduces the monotony associated with traditional imagery training, potentially enhancing both training quality and athletic outcomes.

To systematically examine these effects, the following hypotheses were formulated:

*H1:* The VRBI (Virtual Reality-Based Imagery) model leads to significantly higher muscle activation compared to the VMBR+VM (Visual Motor Behavior Rehearsal + Video Modeling) model.

*H2:* The VRBI model enhances kinesthetic motor imagery skills more effectively than the VMBR+VM model.

*H3:* Athletes using the VRBI model reach peak performance in a shorter time compared to those using the VMBR+VM model.

*H4:* Both VRBI and VMBR+VM models contribute to muscle activation and imagery skill development compared to a control group, but the VRBI model shows superior results.

## Materials and methods

### Location of the research implementation

The research was conducted in the laboratories of the Atatürk University Sports Sciences Application and Research Center.

### Research design

The study investigates the effects of VMBR+VM and VRBI training on athletes’ muscle activation levels using a quasi-experimental design with repeated measures, including VMBR+VM, VRBI, and control groups. Due to the inherent challenges in establishing a neutral sampling process in psychosocial fields, quasi-experimental designs are often preferred within experimental frameworks. A key distinction of quasi-experimental design from true experimental design is that the sample is not randomly selected (McMillan and Schumacher, 2010). To ensure comparability, athletes participating in the study were selected based on similar career stages and years of experience in sports. Athletes from the targeted sports discipline were then randomly assigned to the VMBR+VM, VRBI, and control groups. The dependent variable of the research is the muscle activation levels of the athletes, while the independent variable consists of the imagery training interventions aimed at influencing muscle activation levels.

All measurement tools used in this study were validated and demonstrated high reliability for the athlete population involved. Reliability analyses were conducted to ensure the consistency and accuracy of the data collected, confirming that these tools are appropriate for assessing muscle activation and kinesthetic motor imagery skills in elite athletes.

Additionally, the VRBI training model and related measurement tools have been previously tested and validated for performance assessment in elite athletes. The effectiveness of these models was confirmed in a prior study published in a reputable journal, highlighting their reliability and applicability in sports performance research (Bedir and Erhan, 2021).

### Participants

Since the study aimed to measure muscle activation, participants were purposefully selected from bodybuilders and fitness athletes who demonstrate a high sense of movement awareness, have internalized the sensory aspects of the exercises, and are well-versed in the correct execution of specified movements. This selection ensured the inclusion of individuals with superior strength and power motor skills relevant to the skill being assessed.

The study included licensed athletes actively competing in bodybuilding and fitness disciplines. Eligible participants had prior competitive experience and had consistently trained for at least 3 days per week over the past 5 years. These criteria ensured a homogeneous sample with sufficient experience to engage effectively in imagery training. Participants with psychological or neurological disorders, a history of head trauma, or those using medications affecting the central nervous system or cognitive functions were excluded to prevent potential confounding effects. To maintain standardization across groups, all athletes followed identical resistance training programs throughout the study period. Additionally, the sample size was determined using G*Power analysis to ensure adequate statistical power for meaningful comparisons.

According to the analysis, for the Two-Way ANOVA with Repeated Measures (3×13) at a 90% power level [Power (1 − *β* err prob)] (Brunner et al., 2002; Benish et al., 2017), a 99% confidence interval [*α* err prob.] (Pontillo et al., 2010; Chen and Rappelsberger, 1994), and an effect size [Effect size f] of 0.25, a minimum of 27 samples was determined to be necessary. In anticipation of potential dropouts during the process, a total of 39 voluntary athletes (20 males and 19 females) were included in the study. After removing faulty and incomplete data, analyses were conducted using data from a total of 30 athletes.

In studies related to mental training, it is often observed that applications are conducted with small sample groups (Smith and Holmes, 2004; Buck et al., 2016; Wright and Smith, 2009; Kosteli et al., 2019). The need to conduct separate mental exercises for each athlete participating in the study contributes to this limitation by creating a significant workload.

### Assignment of participants to groups

Participants were randomly assigned to one of three groups: VRBI, VMBR+VM, or the control group, using a computer-generated randomization sequence to minimize selection bias and ensure an equal distribution of participants across groups: Experiment 1 (VMBR+VM, *n* = 13), Experiment 2 (VRBI, *n* = 13), and Control (*n* = 13). In addition to performing the leg extension exercise used in the experimental groups, the control group was exposed to informative videos on nutrition and healthy living to mitigate the placebo effect. This approach ensured that all groups had comparable experiences, while also allowing for the assessment of the specific effects of the training interventions on muscle activation levels.

### Data collection tools

The data collection instruments used in this study consist of three components: The Movement Imagery Questionnaire, Surface Electromyography (sEMG) Measurements, and a Semi-Structured Interview Form. These instruments were selected to comprehensively assess the effects of the training interventions on participants’ mental imagery skills, muscle activation levels, and subjective experiences related to the training process.

### Movement imagery questionnaire—revised MIQ-R

The Movement Imagery Questionnaire was developed by Hall et al. (1985) to assess individuals’ imagery ability. It was subsequently revised and simplified by Gregg et al. (2011), resulting in a scale consisting of a total of eight items that measure four visual and four kinesthetic imagery abilities. The scale includes four movements, which are evaluated in both kinesthetic and visual imagery sub-dimensions. Each item comprises three stages:

- In the first stage, participants are asked to stand in a starting position.
- In the second stage, they are instructed to perform one of four simple motor movements.
- In the final stage, participants are required to visualize the starting position and then imagine either ‘seeing’ or ‘feeling’ the movement without actually performing it.

After completing the imagery process, participants rate the ease or difficulty of imagining the movement on a scale from 1 (easy) to 7 (difficult). The reliability values of the scale are as follows: for the visualization sub-dimension, the reliability coefficient is 0.89, and for the feeling (kinesthetic) sub-dimension, it is 0.88 (Gregg et al., 2011). The scale was adapted into Turkish by Akkarpat (2014). Since kinesthetic motor imagery training conducted in this study, only the kinesthetic imagery sub-dimension of the relevant scale utilized.

### Surface electromyography (sEMG) measurements

#### Muscle activation measurements

Muscle activation measurements were conducted using surface EMG (Noraxon USA, Inc., Scottsdale, AZ). Electrodes were placed on the designated muscles according to the Seniam protocol (SENIAM Project 2005). For rectus femoris (RF), it has been placed in the area that corresponds to 50% (1/2) of the distance between the Anterior Superior Iliac Spine and the Superior Patella. For pectoralis majör (PM) which is control muscle, it has been placed in the upper, middle, and lower sections at a 50% interval between the anterior of the acromion and the sternum. Before all EMG measurements, participants were prepared by lightly shaving their skin in the desired areas where the electrodes would be placed and then wiping it with alcohol-soaked cotton, ensuring stable electrode contact and low skin impedance. After skin preparation, self-adhesive disposable silver/silver chloride pre-gelled dual snap surface bipolar electrodes (Noraxon USA, Inc., Scottsdale, AZ) with a diameter of 1 cm and an inter-electrode distance of 2 cm were attached parallel to the fiber direction of the rectus femoris (RF) and Pectoralis Major (PM). All EMG signals were acquired at 1000 Hz with a bandwidth setting of 5 to 500 Hz (fourth-order Butterworth filter). All raw EMG signals were rectified, integrated, and smoothed using root mean square (RMS) with a 50-millisecond window.

### Process

#### Preparation of 2D/3D videos

For the performance video recording process, a GoPro Fusion 360 camera capable of shooting high-resolution (5.2 K) 360-degree videos was used. To enable the athlete to perform imagery from an internal perspective, the camera was mounted on the athlete’s head using a device. This way, the viewer can imagine themselves in the video. The videos were recorded in the gym where the athlete performed weight training, covering the entire duration of the relevant movement from start to finish. The performance scenarios in which the selected athlete’s avatar was used consisted of the leg extension exercise. The movement was repeated for a total of four sets, with 12 repetitions in each set (4×12). The recorded videos were prepared in 2D video format for the VMBR+VM group in computer format and in 3D video format integrated with virtual reality goggles for the VRBI group using GoPro Fusion Studio, Adobe Photoshop, Premiere, and After Effects programs.

#### Preparation of progressive muscle relaxation scenario

The progressive muscle relaxation scenario for athletes was prepared with the support of two academics major in sports psychology. Before the video modeling, athletes were instructed to perform relaxation exercises following the prepared audio and visual progressive muscle relaxation instructions.

#### Preparation of the imagery scenario

The imagery scenario was prepared with the support of two academics major in sports psychology. The imagery scenarios, developed jointly for each group (VMBR+VM and VRBI), were structured according to kinesthetic motor imagery and the PETTLEP design, focusing on the leg extension movement.

#### Pre-test, mid-test, and post-test measurements

Before the research, participants from the designated sports branch were interviewed to obtain signed voluntary consent forms. Initially, pre-test measurements were taken from participants to assess kinesthetic motor imagery ability (using the Imagery Ability Scale) and yEMG pre-measurements during the imagery task. After the necessary intervention program was implemented, post-test measurements for imagery ability and muscle activation were taken to complete the measurement process.

Additionally, to monitor changes in kinesthetic motor imagery ability and muscle activation throughout the process and to examine the time taken to reach peak performance, all groups underwent mid-test measurements weekly.

### Implementation of intervention programs

#### Visual motor behavioral rehearsal (VMBR) + video modelling (VM)

**Figure 3:**
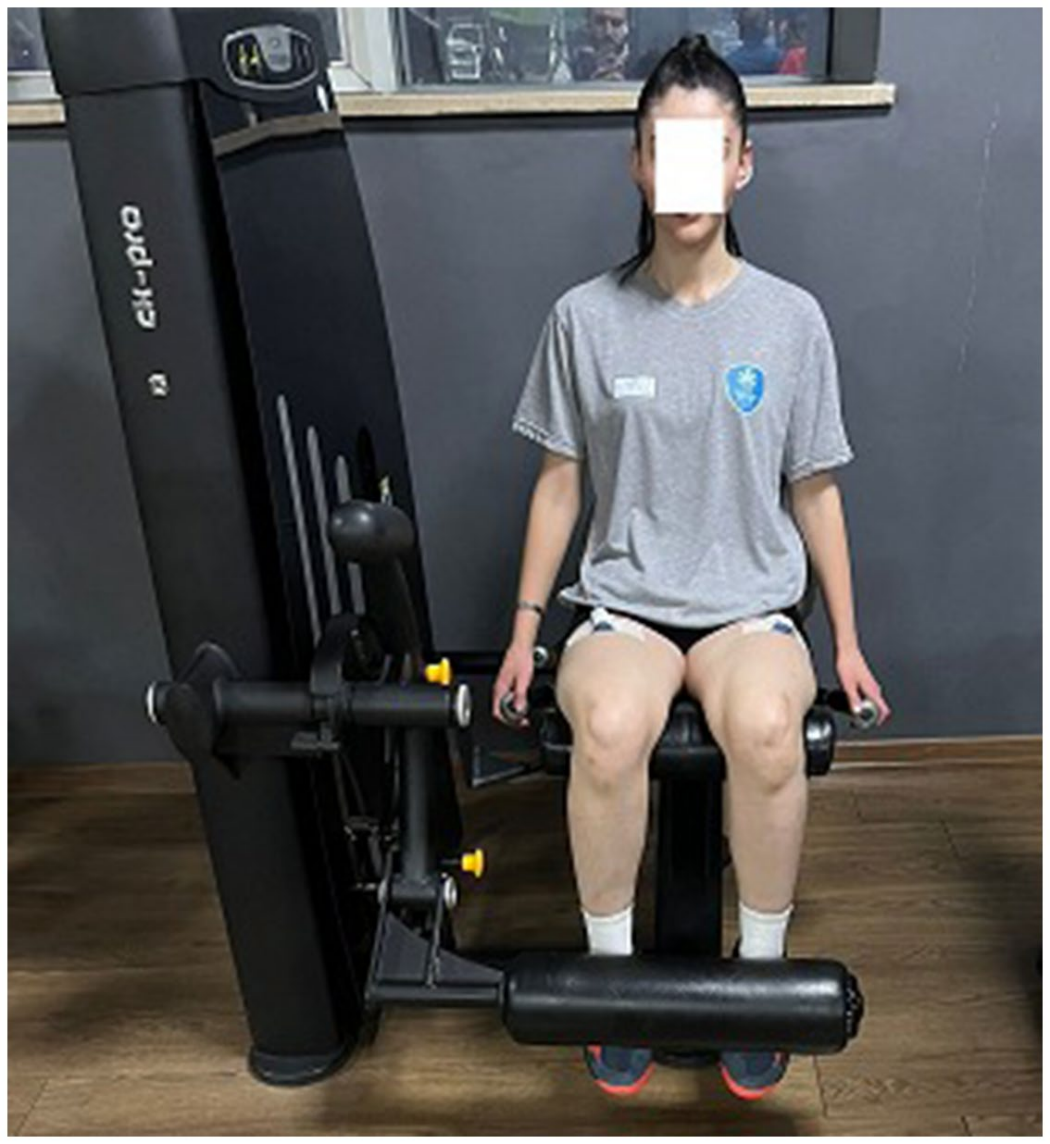
Leg extension.

In Experiment 1, a training program based on the VMBR model developed by Suinn (1972a,b) was implemented, which integrates both visual imagery and relaxation techniques. The VMBR training consists of three stages: (I) relaxing the athlete’s body through a brief version of Jacobson’s progressive relaxation technique (Dass, 1986; Saari and Isa, 2019), (II) viewing 2D performance videos on a tablet (Bedir and Erhan, 2021), (III) guiding the athlete to visualize the leg extension exercise in their mind according to the previously prepared imagery guidelines under the instructions of an expert academic, and (IV) asking the athlete to physically perform the movements they have visualized (see Figure 3).

#### Virtual reality-based imagery model (VRBI)

**Figure 4:**
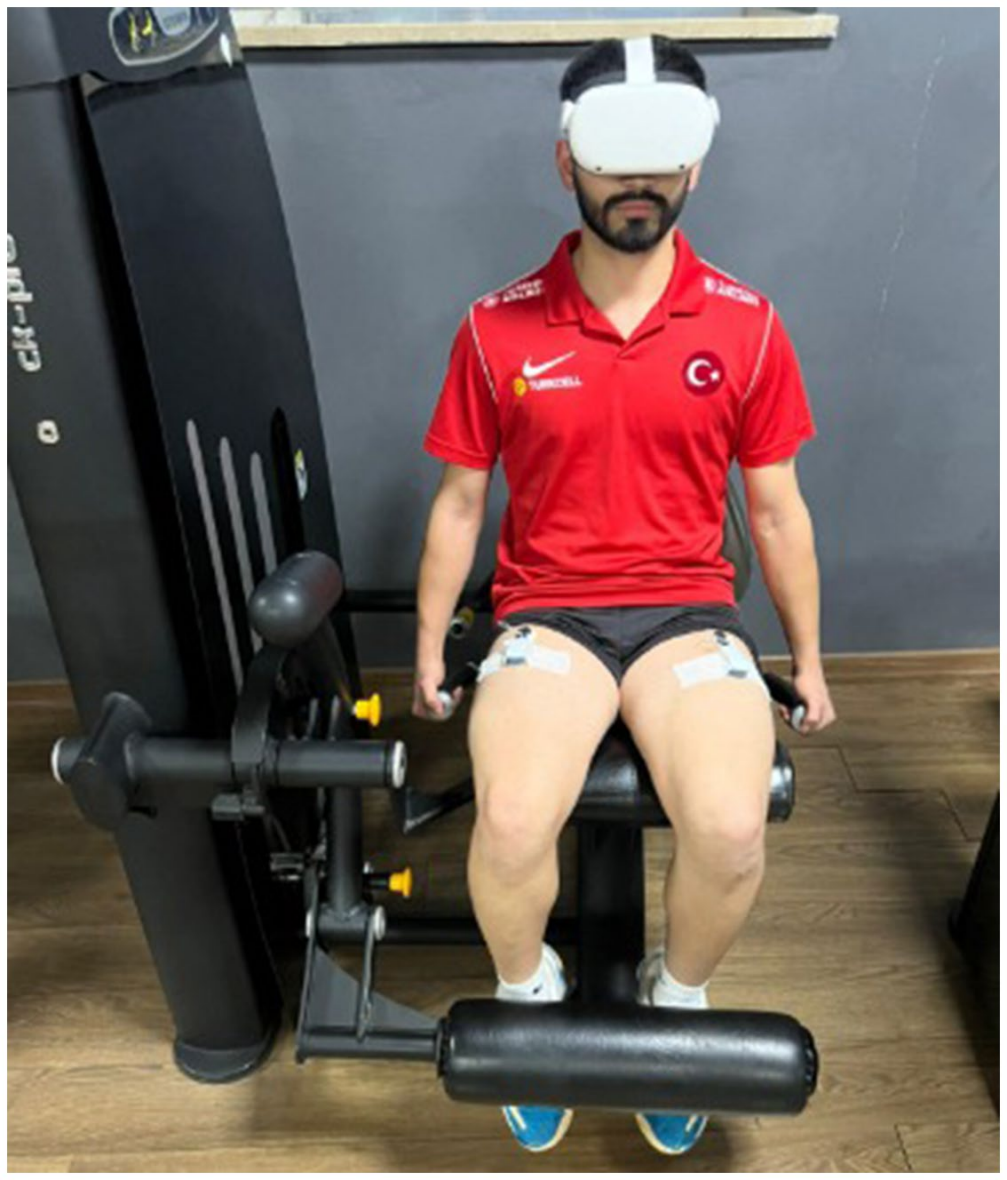
Virtual reality goggles (https://www.meta.com/quest/products/quest-2).

In Experiment 2, the VRBI training program was implemented. Developed by the research team, the VRBI program allows for the entire imagery training to be conducted in a virtual reality environment using VR goggles. The program fundamentally consists of the following stages: (I) Progressive relaxation, (II) Watching a 3D performance video through the virtual reality goggles, (III) Performing imagery guided by recorded imagery instructions, and (IV) Physically executing the performance observed in the video. The Oculus Quest 2 virtual reality goggles were used in this model (Bedir and Erhan, 2021) (see Figure 4).

The videos prepared in 2D and 3D are approximately 10 min long, and during each training session, all four sets were shown to the athletes in the relevant experimental group. To minimize the influence of environmental stimuli on the athletes, the videos were viewed using headphones in a quiet and empty room. Both experimental groups underwent a 25-min intervention program 3 days a week for a total of 12 weeks. The timing of the imagery training sessions was organized according to the athletes’ training schedules. The selection of a 12-week intervention period was based on the observed muscle activation patterns and supported by existing literature on strength and hypertrophy training protocols. Research indicates that significant neuromuscular adaptations and strength gains typically occur within 8 to 12 weeks of consistent training, with performance improvements reaching a plateau beyond this period (Schoenfeld, 2010; Kraemer and Ratamess, 2004).

In our study, muscle activation levels showed a steady increase during the initial weeks, peaking around the 6th to 8th weeks. After this peak, a plateau phase was observed, particularly in the VRBI group, where further gains stabilized despite continued training. This plateau suggests that the neuromuscular system had adapted to the training stimulus, and additional gains would likely require modifications to the training protocol.

Therefore, the 12-week duration was selected to capture both the period of rapid adaptation and the subsequent stabilization phase, providing a comprehensive understanding of the VRBI model’s effects. Terminating the intervention at this point ensures that the results reflect the peak performance achieved and the onset of the plateau phase, optimizing the validity of our findings regarding the impact of imagery-based training on muscle activation.

#### Nutrition and healthy living videos

To minimize the placebo effect in the control group participants, informative videos on nutrition and healthy living were shown for the same duration as the imagery training in the experimental groups.

### Data analysis

#### Analysis of sEMG signals

In the analysis of the EMG data obtained from the individuals participating in the study, the raw data were initially filtered using a high-pass filter. The cutoff frequency for the high-pass filter was set to 5 Hz based on the data density. After the filtering process was completed, the data were smoothed. The Root Mean Square (RMS) method was used for smoothing, with a window interval set to 100 ms. Following the filtering and smoothing processes, the MVC levels specified for each muscle were included in the analysis for evaluation.

#### Analysis of quantitative data

The obtained data were first subjected to preliminary control, during which missing or erroneous data were excluded. Subsequently, normality analysis was conducted by checking Skewness-Kurtosis values, Shapiro–Wilk test results, histograms, and Q-Q plots. Since the data showed normal distribution, a parametric test, Two-Way ANOVA for Repeated Measures (3×13), was performed. The significance level for all results was set at *p* < 0.05. The data analysis was conducted using SPSS version 25 software.

## Results

As shown in Table 1, the weekly variations in the control group of athletes did not show significant changes; however, an increase in the average RF activation was observed in the VRBI and VMBR+VM groups.

**Table 1:** Weekly values of rectus femoris (RF) muscle activation of athletes participating in the study by groups.

Group	VRBI		VMBR + VM		Control	
	$\bar{x}$	ss	$\bar{x}$	ss	$\bar{x}$	ss
1. Week	1.498	0.045	1.078	0.063	0.907	0.120
2. Week	1.522	0.037	1.143	0.057	0.866	0.083
3. Week	1.796	0.071	1.400	0.038	0.879	0.105
4. Week	1.879	0.070	1.437	0.065	0.915	0.097
5. Week	3.316	0.297	2.041	0.115	0.892	0.147
6. Week	4.011	0.522	2.231	0.209	0.846	0.120
7. Week	4.419	0.277	2.491	0.187	0.842	0.074
8. Week	4.336	0.299	2.635	0.202	0.850	0.110
9. Week	4.228	0.176	2.429	0.177	0.883	0.089
10. Week	4.311	0.286	2.573	0.219	0.838	0.089
11. Week	4.174	0.161	2.369	0.157	0.879	0.078
12. Week	4.078	0.710	2.388	0.230	0.855	0.106

The results of the two-way (3 × 12) ANOVA for repeated measures regarding whether the weekly changes in RF activations among the athletes divided into three different groups show significant differences are presented in Table 2.

**Table 2:** ANOVA results for RF muscle activation of the athletes participating in the study.

Source of variance	ss	df	MS	*F*	*p*
Between groups	356.285	29.000	176.911		
Group	353.628	2.000	176.813	1796.608	<0.001
Error	2.657	27.000	0.098		
Within groups	217.281	104.079	46.817		
Measurement (pre-post T)	118.606	3.469	34.187	268.697	<0.001
Measurement × Group	86.757	6.939	12.503	98.272	<0.001
Error	11.918	93.671	0.127		

**Figure 5:**
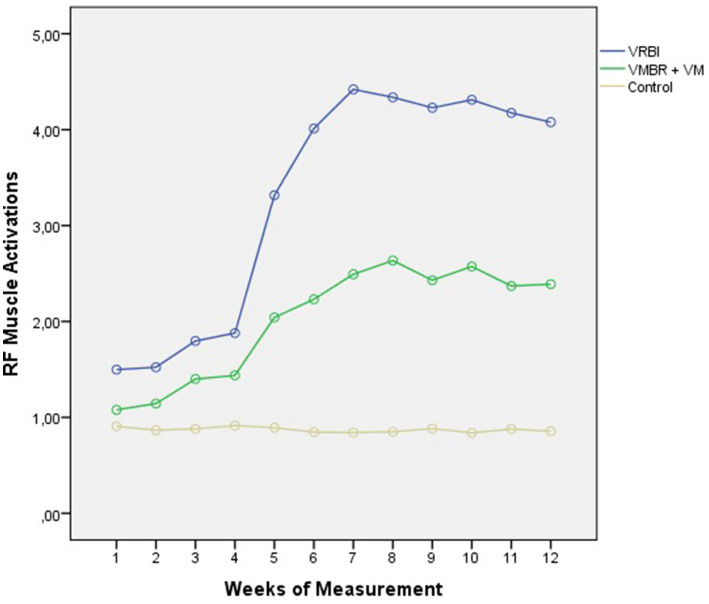
Weekly results graph of RF muscle activations by groups.

As seen in Table 2, there is a significant difference in the average RF muscle activation among athletes in the VRBI, VMBR+VM, and control groups [*F* (2,27) = 1796.608; *p* < 0.01]. Regardless of the group the athletes were assigned to, the differences in weekly activation measurements were also found to be significant [*F* (3,469; 93.671) = 268.697; *p* < 0.01]. This result indicates that there was an increase in RF muscle activation during the imagery sessions throughout the process. Furthermore, the interaction effect between measurement and group on the muscle activation test results is significant [*F* (6,939; 93.671) = 98.272; *p* < 0.01], suggesting that the different imagery training programs applied to the athletes had a significant effect on their muscle activations (see Figure 5).

**Figure 6:**
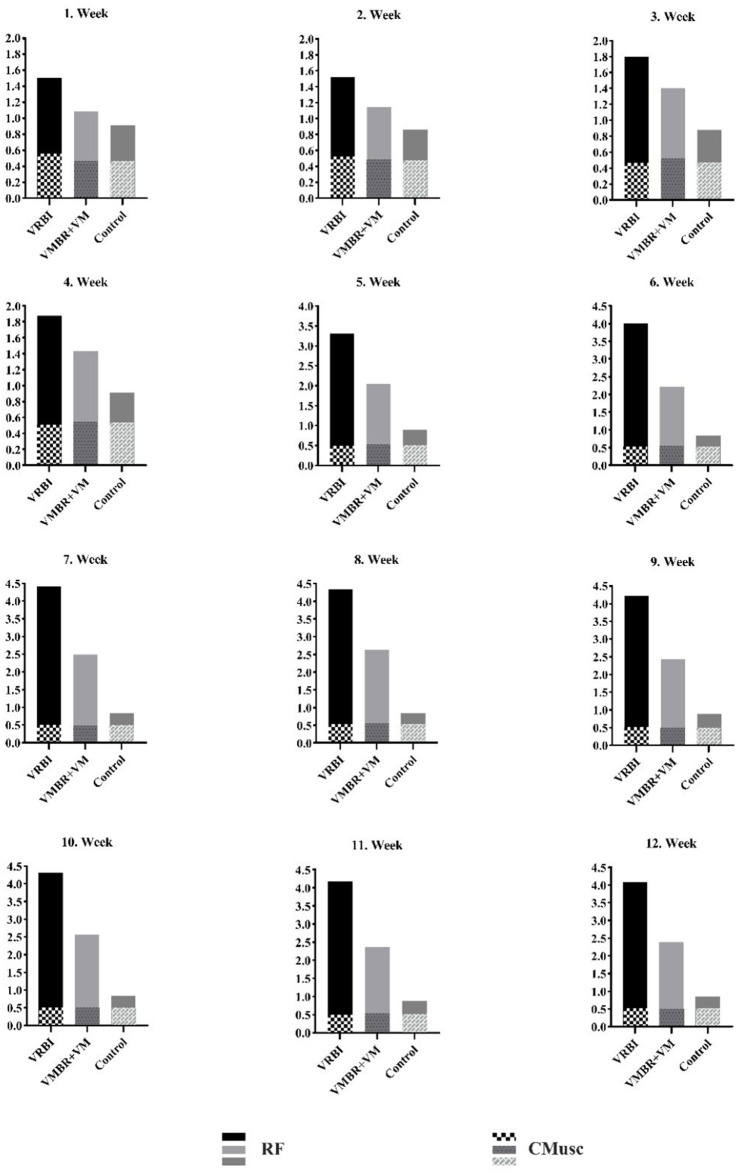
Activation graph of RF muscle activations by groups and the control muscle group.

Figure 1 graphically illustrates the changes in Rectus Femoris (RF) muscle activations among the groups throughout the experimental process. Upon examining the figure, it can be observed that, at the end of the first week, the muscle activations of athletes in the VRBI and VMBR+VM groups exhibited a relatively slow increase after the second week, with this increase particularly accelerating by the end of the fourth week. Muscle activation in the VRBI group continued to rise until the end of the seventh week, but from the eighth week onward, this increase plateaued, indicating a stabilization in muscle activation. In contrast, athletes in the VMBR+VM group reached their peak activation by the end of the eighth week. No significant differences in muscle activations were noted among the athletes in the control group. The results suggest that the athletes receiving VRBI training achieved peak activation 1 week earlier than those in the VMBR+VM group, and according to the Psycho Neuromuscular Theory, it can be inferred that the time period of the highest Carpenter effect is during the seventh week (see Figure 6).

Activation data were collected from all participants for the Rectus Femoris (RF) muscle used during imagery, as well as the control muscle group identified as the Pectoralis Major (PM) muscle. Upon examining Figure 2, it is observed that the control muscle remained constant across all weeks, and the results of the conducted pairwise comparison analyses revealed significant differences between the VRBI, VMBR+VM, and control groups across all weeks.

Upon examining Table 3, it is observed that the pre-test scores of the athletes in the control and experimental groups for kinesthetic motor imagery skills are quite similar, while differences are evident in the post-test scores compared to the control group.

**Table 3:** Mean and standard deviation values of pre- and post-test scores of kinesthetic motor imagery skills by groups for the athletes participating in the study.

Group	Pre test		Post test	
	$\bar{x}$	ss	$\bar{x}$	ss
VRBI	1.75	0.44	6.42	0.28
VMBR + VM	1.77	0.32	4.50	0.37
Control	1.37	0.17	1.80	0.28

**Figure 7:**
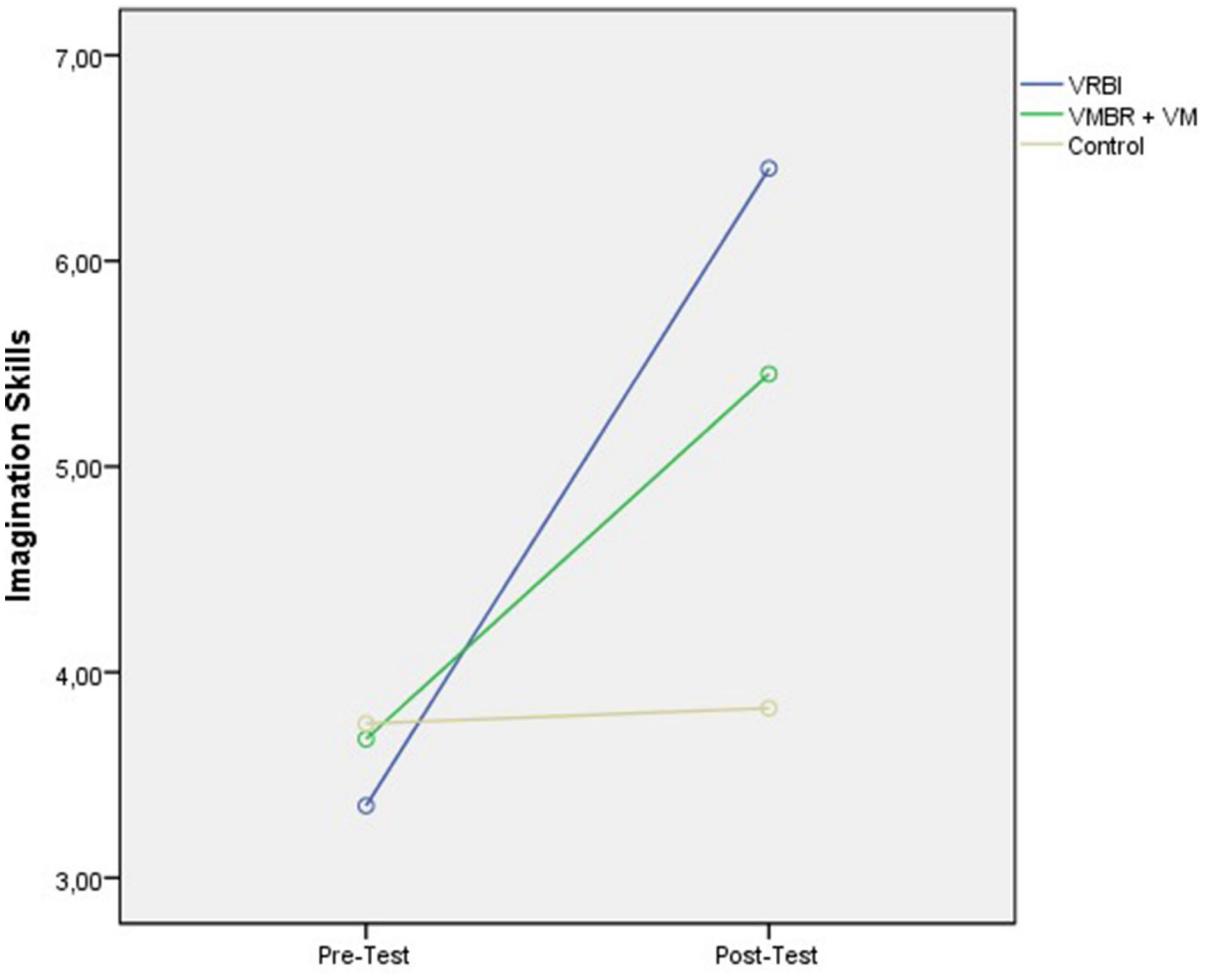
Results graph of pre- and post-test scores for kinesthetic motor imagery skills by groups.

Upon examining Table 4, significant differences in kinesthetic motor imagery scores were found among the athletes in the VMBR+VM, VRBI, and Control groups [*F* (2,27) = 295.579, *p* < 0.05]. Regardless of the group in which the participants were in, a significant difference was found between the pre-test and post-test scores for kinesthetic motor imagery [*F* (1,27) = 219.434, *p* < 0.05]. This result indicates that the intervention applied during the process increased the athletes’ ability to perceive imagery. As shown in the table, the measurement × group interaction effect on the athletes’ imagery perception scores was significant [*F* (2,27) = 2.784, *p* < 0.05]. Therefore, it can be stated that the imagery training applied to the athletes led to differentiation in visualization ability scores among the groups (see Figure 7).

**Table 4:** ANOVA results of kinesthetic motor imagery scores of athletes participating in the study.

Source of variance	ss	df	MS	*F*	*p*
Between groups	66.609	1	139.85		
Group	63.700	2	31.850	295.579	<0.000
Error	2.909	27	108		
Within groups					
Measurement (pre-post T)	102.051	1	102.051	219.434	<0.000
Measurement × Group	45.258	2	22.629		
Error	2.784	27	0.103		

Upon examining Table 5, no significant differences were observed in rectus femoris muscle activation between genders across the groups.

**Table 5:** Activation results of RF muscle by groups and the gender.

Gender	SGTİ		VM		Control		*p*
	$\bar{x}$	ss	$\bar{x}$	ss	$\bar{x}$	ss	
Male	3.343	0.039	1.966	0.050	0.850	0.036	0.704
Female	3.253	0.039	2.041	0.033	0.903	0.044	

## Discussion

The aim of this study is to examine the effects of the VRBI training model on athletes’ muscle activation and kinesthetic motor imagery skills within the framework of the psycho-neuro-muscular theory, and to compare it with the popular approach used in contemporary imagery training, VMBR+VM.

The findings indicate that the newly developed imagery model, VRBI, yielded more favorable results in terms of muscle activation compared to the most frequently and popularly used imagery model, VMBR+VM. Additionally, it was observed that both groups exhibited significant differences in activation levels compared to the control group across all weeks. Another important result of the study is that athletes in the VRBI group adapted to the process more quickly, resulting in a rapid increase in their muscle activation levels.

In the study, the real effect of imagery was attempted to be understood by using not only the target muscle group but also the control muscle group variable. Activation data were collected from all participants for the target muscle used during imagery, namely the Rectus Femoris (RF), as well as the control muscle group identified as the Pectoralis Major (PM). The findings revealed that the control muscle remained almost constant across all weeks, and the results of the pairwise comparison analyses indicated significant differences between the VRBI, VMBR+VM, and control groups throughout all weeks. Finally, it was observed that the athletes in both experimental groups improved their imagery skills compared to the control group throughout the intervention program. Furthermore, significant differences between the groups in terms of both activation and kinesthetic motor imagery skills highlighted the advantages of the VRBI group’s model.

While the muscle activations of the athletes in the VMBR+VM group showed a relatively slow increase after the second week, this increase notably accelerated by the end of the fourth week. In contrast, the muscle activation of the athletes in the VRBI group continued to rise until the end of the seventh week, after which the increase plateaued starting from the eighth week. The athletes in the VMBR+VM group reached their peak activation by the end of the eighth week. No significant differences were observed in the muscle activations of the athletes in the control group. The results suggest that the athletes receiving VRBI training achieved peak activation 1 week earlier than those in the VMBR+VM group, and according to the psycho-neuro-muscular theory, the time period with the highest Carpenter effect is indicated to be the seventh week.

These results indicate that both imagery interventions were effective and beneficial. In the study, the advantages of the VRBI model in terms of muscle activation and kinesthetic motor imagery skills are thought to arise from its greater alignment with the PETTLEP model within the framework of the functional equivalence hypothesis. It is believed that incorporating multiple sensory modalities into the imagery training contributed to this effect. According to the functional equivalence hypothesis, the mental representation of movement during motor imagery activates similar brain areas as those engaged during actual motor execution, thereby strengthening the memory pathways related to the motor task (Harris and Hebert, 2015). Some studies have found that interventions based on the PETTLEP model, which maximally utilize functional equivalence, have shown positive effects when applied in various sports including athletics, gymnastics, and strength training (Lebon et al., 2010; Wright and Smith, 2007).

Numerous studies support the findings of our research, particularly indicating that imagery training utilizing the PETTLEP model has shown significant improvements compared to traditional imagery methods (Wilkes and Summers, 1984; Tenenbaum et al., 1995; Wright and Smith, 2007).

In a study conducted on bodybuilders, where the PETTLEP model was applied in terms of motor imagery skills, the findings indicated that the imagery intervention designed with the PETTLEP model had a positive impact even on challenging strength tasks (Smith et al., 2020). Effective imagery allows an individual to feel as though they are actually performing the movement while imagining it. The increase in muscle activation levels among the athletes in the VRBI group used in the study can be explained by the model’s provision of an opportunity to experience a sense of real movement through three-dimensional environment videos. VRBI is an acronym for the proposed core elements of an effective imagery intervention based on PETTLEP (i.e., physical, environment, task, timing, learning, emotion, and perspective components); this approach has been demonstrated in various studies to enhance the effects of imagery training by ensuring that the imagery performed closely represents actual movement (Smith et al., 2008, 2020; Wright and Smith, 2009; Battaglia et al., 2014; Anuar et al., 2018).

In the study conducted by Wright and Smith (2009), it was demonstrated that the video-supported PETTLEP imagery method was more effective than traditional imagery methods during a strength task such as the biceps curl (1 repetition maximum, or 1RM). PETTLEP imagery allows individuals to visualize the movement in a more realistic and detailed manner during mental training, leading to enhanced visualization performance. The video support helped athletes see the correct form of the movement, facilitating a clearer mental imagery process. The findings indicated that video-supported PETTLEP imagery was more effective than conventional visualization methods, with athletes in the experimental group experiencing a 10.7% increase in squat performance, while no improvement was observed in the control group. Similarly, our developed Virtual Reality-Based Imagery (VRBI) training advances the core principles of PETTLEP, transforming the imagery process into a real-time and interactive experience. VRBI allows athletes to conduct mental training in a virtual environment, resulting in deeper muscle activation and motor skill development compared to traditional methods. Therefore, it has been observed that VRBI training yields superior performance outcomes in terms of muscle activation compared to PETTLEP and traditional imagery methods.

In a different study, Battaglia et al. (2014) investigated the effects of video modeling-supported imagery on gymnasts’ jumping performance and imagery skills. According to the findings of the study, athletes who utilized a combination of video modeling and imagery methods achieved higher scores compared to the control group, which only engaged in physical training. These athletes demonstrated a significant improvement in both their imagery skills and jumping performance. The results from the literature review indicated that supporting imagery with video modeling enhances the quality and effectiveness of the imagery process (Smith et al., 2007; Buck et al., 2016).

Similarly, in our study, the VMBR+VM group achieved higher scores in terms of muscle activation compared to the control group. However, it was observed that the scores of the VMBR+VM group remained at lower levels compared to the VRBI group. This can be explained by the fact that imagery interventions integrated with virtual reality technologies effectively represent the PETTLEP model. The VRBI model allows for a reduction in the duration of this process while ensuring that the represented skills and movements are encoded in the brain in a clearer and more precise manner.

While the PETTLEP model and video modeling are important factors in determining the effectiveness of imagery, applying imagery alone does not contribute to performance. The key factor for optimally enhancing both imagery skills and performance gains is ensuring that imagery interventions are conducted alongside physical performance (Cumming et al., 2007; Pesce et al., 2007; Burton and Raedeke, 2008; Bedir and Erhan, 2021). Studies utilizing neuroimaging have shown that kinesthetically performing the imagined movement enhances neural activity and contributes to motor imagery (Fadiga et al., 1998; Clark et al., 2004; Buccino et al., 2001). In this study, both the VMBR+VM and VRBI training programs were implemented in conjunction with physical training. As a result, positive outcomes were achieved in terms of muscle activation and kinesthetic motor imagery skills.

It was found that the athletes in the VRBI, VMBR+VM, and Control groups showed significant improvement in both muscle activation levels and imagery skills compared to the control group throughout the intervention program. The pre-test scores for kinesthetic motor imagery skills of athletes in the Control and Experimental groups were similar, while differences between the groups emerged in the post-test scores.

Significant differences in favor of the VRBI group were also observed between the VRBI and VMBR+VM groups. These results indicate that the VRBI imagery intervention programs have a distinct impact not only on muscle activation but also on kinesthetic motor imagery skills. The differentiation of scores from the kinesthetic motor imagery dimension of the movement imagery scale in favor of the VRBI model can be attributed to its effective representation of the PETTLEP model, as well as the advantages of providing a realistic movement experience through imagery training in a three-dimensional environment. Furthermore, the measurement of the imagined movement as a real kinetic movement throughout the assessment process can also be considered another reason for the increase in kinesthetic imagery skills. Im et al. (2016) found that when motor imagery is used with virtual reality support, it enhances corticomotor excitability. In their study, the central nervous system responses of the virtual reality-supported motor imagery group were found to be significantly greater than those of the group engaging in only motor imagery, comparing stroke patients and healthy individuals. In our study, the positive contributions of the VRBI program to athletes’ kinesthetic imagery skills and muscle activation levels are clearly evident and align with the literature.

## Conclusion

The aim of this study is to compare the recently developed VRBI (Virtual Reality-Based Imagery) model with the widely used VMBR+VM (Visual Motor Behavior Rehearsal + Video Modeling) model and to highlight the advantages and validity of the VRBI model in terms of muscle activation, kinesthetic motor imagery skills, and time to peak performance. The findings obtained at the end of the study demonstrate that imagery intervention programs have contributed positively to muscle activation and imagery perception skills in athletes from the VRBI and VMBR+VM groups compared to the control group. Both the 12-week VRBI and VMBR+VM imagery intervention trainings significantly contributed to the development of both muscle activation and imagery skills among athletes. In addition, the superiority of the VRBI imagery training model over other models, especially in terms of muscle activation and kinesthetic motor imagery skills, was confirmed by the study’s findings.

### Recommendations

When planning imagery training aimed at enhancing athletes’ performance, the use of the VRBI model may provide higher efficiency in muscle activation and kinesthetic imagery skills. The increased application of virtual reality-supported imagery studies in sports rehabilitation and performance enhancement processes can contribute to athletes’ physical and mental development. By considering peak performance times in imagery training, optimal training durations can be established. The active use of the VRBI (Virtual Reality-Based Imagery) model in imagery training during rehabilitation processes after injuries may contribute to the re-functionalization of muscles. In particular, allowing injured athletes to mentally sustain their muscle activation without physical exercise can yield beneficial results in preventing atrophy and accelerating the recovery process. Integrating advanced technologies like virtual reality into imagery processes can enhance motivation and make the rehabilitation process more interactive, enabling athletes to regain their physical performance more quickly after an injury.

One of the key advantages of VRBI in clinical settings is its ability to provide controlled, immersive environments that facilitate motor imagery training without physical strain. Patients with mobility restrictions can engage in imagery-based rehabilitation, which may aid in maintaining neuromuscular activation and preventing muscle atrophy. Furthermore, by integrating biofeedback and real-time movement simulation, VRBI can enhance motivation and adherence to rehabilitation programs, which are often challenging for patients undergoing long-term recovery.

Therefore, the regular implementation of VRBI-based imagery training in sports injuries and rehabilitation programs can help preserve and develop muscle memory and motor skills. To evaluate the effectiveness of the VRBI model in rehabilitation processes post-injury, controlled experiments should be conducted on an appropriate sample consisting of athletes. In this context, the sample group can be selected from athletes who have experienced injuries in the lower extremities (knee, ankle) or upper extremities (shoulder, elbow). Individuals in the sample group should be homogeneous in terms of injury severity and duration, while selecting athletes from different sports can allow for the observation of the model’s broad-spectrum effects.

The study’s findings suggest that while VRBI has strong applications for individual sports, its potential for team sports should not be overlooked. It is recommended to explore how VRBI can simulate team-based scenarios, enabling athletes to rehearse strategic plays and improve group coordination. Future research should focus on adapting VRBI technology to reflect the complex dynamics of team sports and evaluating its effectiveness in such settings.

### Limitations

This study provides valuable insights into the effects of virtual reality-based imagery (VRBI) and mental training on rectus femoris muscle activation; however, several limitations should be noted:

The study was conducted with a relatively limited sample size, which may restrict the generalizability of the findings to broader populations. Future research should aim to include a larger and more diverse participant group to strengthen the external validity of the results. The study specifically focused on the rectus femoris muscle, leaving the effects of VRBI on other muscle groups unexplored. Investigating additional muscle groups, including those with synergistic or antagonistic roles, could provide a more comprehensive understanding of VRBI’s impact on neuromuscular activation. While the study demonstrates the effectiveness of VRBI in individual-based exercises, its applicability to team sports remains untested. Future studies should explore how VRBI can be adapted to team-based scenarios, such as strategic decision-making and coordination exercises, to evaluate its broader utility in sports settings.

## Data Availability

The datasets presented in this study can be found in online repositories. The names of the repository/repositories and accession number(s) can be found below: DOI: 10.48623/aperta.273958, https://aperta.ulakbim.gov.tr/record/273959.
