# Supplementary figures and images for: Investigation of the effect of a virtual reality-based imagery training model on muscle activation in athletes
Source: Front Psychol. 2025 Feb 27;16:1553327. doi: 10.3389/fpsyg.2025.1553327 (PMC11905993; doi:10.3389/fpsyg.2025.1553327)

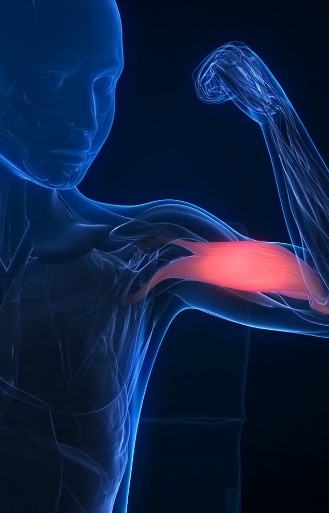

Supplement: Supplementary file 1 [file Image_1.jpeg]

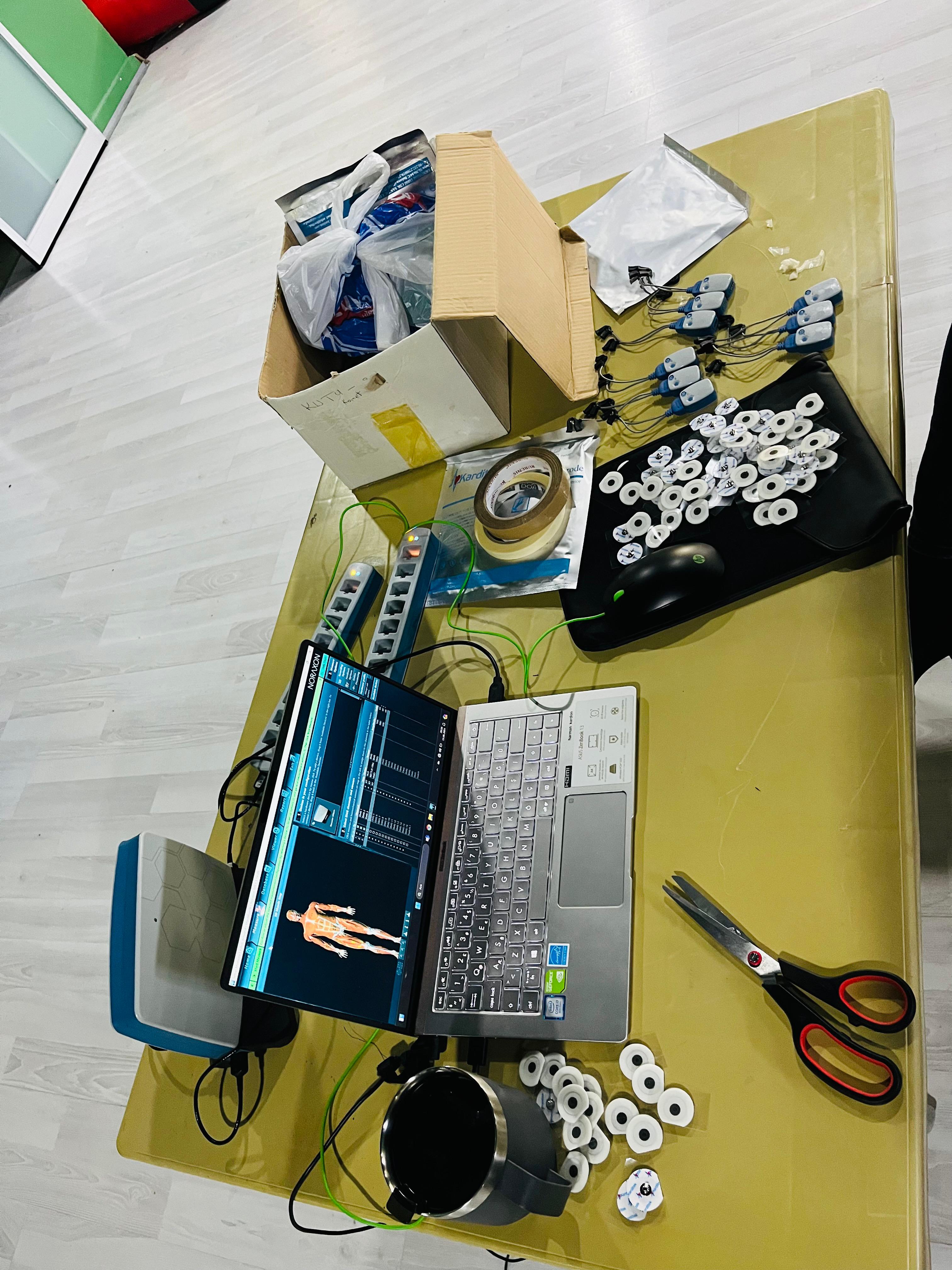

Supplement: Supplementary file 2 [file Image_2.jpeg]
